# Nutrition aspects in children receiving maintenance hemodialysis: impact on outcome

**DOI:** 10.1007/s00467-007-0728-3

**Published:** 2009-05-01

**Authors:** Poyyapakkam R. Srivaths, Craig Wong, Stuart L. Goldstein

**Affiliations:** 1 Department of Pediatrics, Baylor College of Medicine and Renal Services, Texas Children’s Hospital, 6621 Fannin Street, MC 3–2482, Houston, TX 77030 USA; 2 Department of Pediatrics, Section of Pediatric Nephrology, University of New Mexico Health Sciences Center, Albuquerque, NM USA

**Keywords:** Malnutrition, Maintenance hemodialysis, nPCR, Children, IDPN

## Abstract

Children with end-stage renal disease (ESRD) have rates of mortality estimated to be 30-times higher than expected for age compared with those of healthy children. Physical manifestations of under-nutrition, such as body mass index (BMI) and low height standard deviation score (SDS), have been associated with increased risk of mortality. Traditional measures, such as height, weight and serum albumin concentration, may not be accurate indicators to assess the nutritional status of children receiving maintenance hemodialysis. Normalized protein catabolic rate (nPCR) has emerged as a better marker of nutritional status of such children. Meeting the special nutritional needs of these children often requires nutritional supplementation, by either the enteral or the parenteral route. Recently, in children receiving maintenance hemodialysis who are malnourished, intradialytic parenteral nutrition (IDPN) has been utilized as a means to provide additional protein and calories. This article is a state-of-the-art review of malnutrition in children receiving maintenance hemodialysis, with special focus on outcome, nPCR and IDPN.

## Malnutrition

### Background

#### Malnutrition vs cachexia

Malnutrition is a complex concept that is difficult to define. The World Health Organization defines malnutrition as the “term used to refer to a number of diseases, each with a specific cause related to one or more nutrients and each characterized by cellular imbalances between supply of nutrients and energy on one hand and the body’s demand for them to ensure growth, maintenance and specific functions on the other hand.” [1, 2]. Inadequate nutrient intake has traditionally been thought to be the most important single cause of malnutrition in patients receiving maintenance hemodialysis (HD) [3]. However, since provision of nutrients alone does not correct this state, Mitch and others have questioned the use of this term for patients with end-stage renal disease (ESRD) that are receiving maintenance dialysis [4–7]. They propose the term ‘cachexia’ to define the nutritional state in ESRD, with factors such as metabolic acidosis and inflammation causing muscle wasting and protein catabolism by involvement of the ubiquitin–proteosome pathway.

Malnutrition [defined either by body mass index (BMI) or biochemical markers such as albumin] is a well-recognized complication and an independent risk factor for increased mortality in adults with ESRD receiving maintenance HD [8, 9]. Children with ESRD have rates of mortality approximately 30-times higher than expected for age when compared with those of healthy children [10, 11]. Although multiple factors might be responsible, physical manifestations of malnutrition, such as short stature, lower BMI, in the pediatric population have been demonstrated to be associated with increased risk of death.

As the definition and assessment of malnutrition have not been standardized, determination of the exact prevalence of malnutrition in children receiving hemodialysis is difficult. Height standard deviation score (SDS) and weight SDS, available from the North American Pediatric Renal Transplant Cooperative Study (NAPRTCS) 2006 annual data, reveal mean height standard deviation score at initiation of hemodialysis to be −1.50, declining to −1.93 after 24 months, and weight SDS to be −1.00 at initiation, declining to −1.55 after 24 months [12], though low height SDS can be due to reasons other than poor nutrition (see Table 1).

**Table 1:** Nutritional assessment parameters and their limitations (*DEXA* dual-energy X-ray absorptiometry)

Parameter	Limitation
Weight	False elevation due to fluid overload
Height	Influenced by alterations of growth hormone axis, delayed puberty, renal osteodystrophy, independent of nutritional state
MAC and triceps	Can be influenced by altered regional distribution of fat/muscle
Skin fold thickness	
Bio-impedance	Unpredictable effect on estimation of fat-free mass from total body water in renal failure
Albumin/pre-albumin	Albumin level associated with outcome, but levels influenced by inflammation and fluid overload; also limited by long half-life. Pre-albumin shorter half-life but influenced by inflammation and fluid overload
Bone density (DEXA)	Fluid overload can cause overestimation of lean mass
Normalized protein catabolic rate (nPCR)	Only few studies available but emerging as nutrition marker for children receiving HD

*MAC* mid arm circumference

##### Outcome

Few large-scale studies have assessed the impact of malnutrition on long-term outcomes in children receiving long-term dialysis. Wong et al. [13] investigated the association between height, weight, growth velocity, BMI and mortality in 1,949 children with ESRD, including those receiving HD, peritoneal dialysis or with a kidney transplant. Their 7-year review of the US Renal Data System database documented each decrease in height SDS of 1, increased adjusted mortality risk (aRR) by 14% (aRR, 0.88, 95% CI 0.79 to 0.98). In addition, each decrease in growth velocity of 1 SDS increased mortality risk by 12% (aRR 0.89, CI 0.80 to 1.00). The association between height SDS, growth velocity SDS and mortality held true across all ages and was independent of ESRD treatment modality. Subsequently, Furth et al. [14], in a separate study, demonstrated increased risk of death (RR 2.9, 95% CI 1.6, 5.3) in children with severe growth failure (≤ 3 SDS) when compared with children with normal growth.

**Fig. 1:**
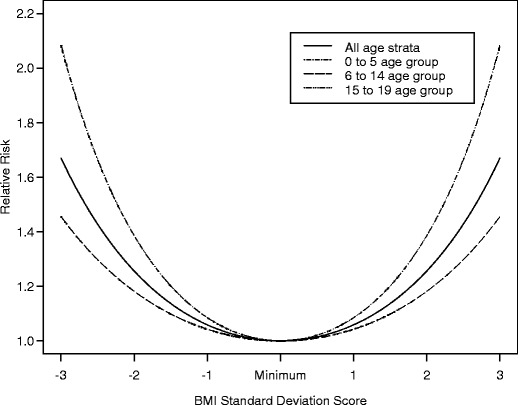
The relative risk of death and confidence intervals for BMI standard deviation scores among children with ESRD. The minimum relative risk of death is observed at a BMI = −0.50 SDS. The U-shaped association was statistically significant; *P* = 0.001. Note that the lines for all age strata and those of the 6 to 14 year age groups overlap. (Used with permission from C. Wong)

**Fig. 2:**
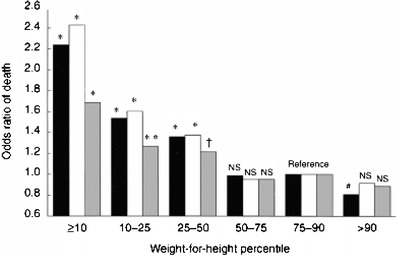
Odds ratio of death among 12,965 adult patients grouped by weight-for-height percentiles. Three representations are given in a cluster of three bars. *Black bars* represent unadjusted odds ratio, *white bars * represent odds ratio adjusted for clinical variables (age, gender, race and diabetes mellitus), *gray bars*, adjusted for clinical and laboratory variables (pre-dialysis serum albumin, creatinine and cholesterol values). *NS* not significant. Reprinted with permission from Macmillan Publishers Ltd., Kidney Int 56:1136–1148 [15]. The *P* values symbols over a bar indicate the significant difference between the values for that bar and for the reference group. ^*^P<0.001, ^**^P=0.11, ^†^*P*=0.21, ^#^*P*=0.038

Multivariate analysis of body mass index SDS in the study by Wong et al. [13] showed a U-shaped association between BMI and risk of death, with extremes in BMI associated with increased risk of death (Fig. 1). The finding of an increased risk of death with a higher BMI in children with ESRD is in contrast to the findings in adults, where increased weight actually improves outcome (Fig. 2). [15]. In fact, some of the traditional protective factors contributing to decreased cardiovascular mortality in the general population, such as low cholesterol level and decreased weight, seem to have a negative influence (reverse epidemiology) on mortality in adults with ESRD [16].

In a separate pediatric study, Wong et al. [17] explored a potential association of serum albumin and mortality over a 3-year follow-up period in 1,723 children with ESRD. There were 93 deaths in 2,953 patient–years of observation. Multivariate analysis revealed that patients with serum albumin < 3.5 g/dl exhibited a 90% greater risk of death than patients with serum albumin > 3.5 (aRR 1.9; 95% CI, 1.16 to 3.10). In addition, age, ESRD treatment modality (transplant patients with lower mortality than those undergoing either hemodialysis or peritoneal dialysis) and height SDS were also independently associated with mortality. Though these studies demonstrated that low height SDS and serum albumin levels are independent risk factors for mortality, both variables may be influenced by several factors other than nutrition status and do not provide mechanistic insight into the associations observed. Perturbation of growth hormone/insulin-like growth factor/growth plate chondrocyte axis in pediatric ESRD is well known and causes growth retardation. The studies by Wong et al. [13] and Furth et al. [14] do not include data on growth hormone use in this group of children, and since growth hormone can increase height SDS in children with ESRD, it can alter the height SDS variable, independent of nutritional status. Serum albumin is not only influenced by nutrition status but also by fluid status and inflammation [18, 19]. Kaysen et al. [19] demonstrated in 64 hemodialysis patients enrolled in the hemodialysis (HEMO) study that inflammation caused increased fractional catabolic rate of serum albumin, causing low levels independent of nutritional variables. Jones and colleagues investigated the effect of serum albumin and hydration in 49 patients on hemodialysis and showed that serum albumin level was independently associated with extra-cellular fluid status [18]. Goldstein and co-workers [20] and Orellana and colleagues [21] found that serum albumin was a poor marker of nutritional status in children with severe malnutrition that were receiving HD and who were being given intradialytic parenteral nutrition (IDPN).

##### Nutrition assessment

For the monitoring of nutritional status of children on maintenance hemodialysis, the Kidney Disease Outcomes Quality Initiative (K-DOQI) recommends the following measures: dietary interview, serum albumin, height or length, estimated dry weight, mid-arm circumference, skin fold thickness, head circumference for children aged 3 years or less, and SDS or Z scores [22]. The pediatric renal dietician is invaluable to assess and manage malnutrition in children with renal disease, and detailed guidelines exist with regard to frequency of nutrition assessment of children on maintenance hemodialysis [23]. However, the above parameters have their limitations [24], as highlighted in Table 1.

The role of normalized protein catabolic rate (nPCR) in the monitoring of the nutritional status of children receiving long-term hemodialysis has been studied recently. Protein catabolic rate (PCR) is a quantitative measure of protein catabolism and can be calculated from nitrogen mass balance studies with measurement of nitrogen excretion in dialysate, urine and feces. However, this is cumbersome and impractical in clinical settings. Borah et al. [25] obtained urea generation rate and PCR (by nitrogen balance studies) separately, and, by the use of regression estimates, elegantly demonstrated the relationship between urea generation rate and protein catabolic rate in five patients receiving long-term HD, paving way for the derivation of PCR from the interdialytic rise in urea generation rate. The urea generation rate can be derived either by formal urea kinetic modeling (UKM) or by an algebraic equation [26]. The latter method has been shown to have excellent correlation with UKM [26]. An estimated urea generation rate (estG) is derived from the difference between the post-treatment blood urea nitrogen (BUN) (C1, expressed in milligrams per deciliter) and pretreatment BUN (C2):

$${\text{estG}}\left( {{{{\text{mg}}} \mathord{\left/ {\vphantom {{{\text{mg}}} {\min }}} \right. \kern-\nulldelimiterspace} {\min }}} \right) = {{\left( {\left[ {C2 \times V2} \right] - \left[ {C1 \times V1} \right]} \right)} \mathord{\left/ {\vphantom {{\left( {\left[ {C2 \times V2} \right] - \left[ {C1 \times V1} \right]} \right)} T}} \right. \kern-\nulldelimiterspace} T}$$

where V1 is post-dialysis total body water (in deciliters; V1 = 5.8 dl/kg × post-dialysis weight in kilograms), V2 is predialysis total body water [in deciliters; V2 = 5.8 dl/kg × predialysis weight in kilograms], and T is time in minutes from the end of dialysis treatment to the beginning of the next treatment. Then, estimated nPCR can be calculated from the modified equation of Borah et al. [25]:

$${\text{nPCR}} = {{5.43 \times {\text{G}}} \mathord{\left/ {\vphantom {{5.43 \times {\text{G}}} {{\text{Vd + 0}}{\text{.17}}}}} \right. \kern-\nulldelimiterspace} {{\text{Vd + 0}}{\text{.17}}}}$$

where Vd represents total body water after dialysis (0.58 × weight in kilograms). We recommend that nPCR be measured monthly, together with Kt/V in dialysis units where Kt/V is assessed.

Cano et al. [27], in an observational study, found significant positive correlation between Kt/V vs daily protein intake (DPI), protein catabolic rate (calculated from measuring nitrogen losses in urine and dialysate) and normalized protein nitrogen appearance (which they derived from urea generation rate) but failed to observe correlation between Kt/V and nitrogen balance in 20 children undergoing long-term peritoneal dialysis. The absence of a relationship between dialysis dose and nitrogen balance suggested that the correlation observed between dialysis dose and protein catabolic rate resulted from a mathematical tautology; however, DPI in their patient population correlated with PCR (r = 0.9), suggesting that PCR was, in fact, reflective of nutritional status. Grupe and colleagues [28] and Harmon and co-workers [29] showed the correlation of nPCR to dietary protein intake about 20 years ago and demonstrated that, without an increase in dialysis dose, a moderate protein intake resulted in a positive nitrogen balance. Recently, three pediatric studies [20, 21, 30] have shown nPCR as a better marker than albumin of subsequent weight loss in children on long-term hemodialysis. Patients in the studies received adequate dialysis (single pool Kt/V of 1.3); however, nPCR varied with nutritional status. An nPCR < 1 g/kg per day was associated with weight and BMI- for-age loss in the subsequent month, whereas nPCR > 1 g/kg per day was associated with progressive increases in both parameters [30]. The relationship of nPCR to Kt/V might be interpreted to reflect the increased dose of dialysis necessary to control the urea of patients ingesting larger quantities of protein [31]. However, nutrition analysis from the HEMO study in adults demonstrated that the increasing of the dialysis dose beyond the standard dose or the use of high-flux dialysis did not improve albumin level or post-dialysis weight [32]. Marsenic et al. [33] showed, in 17 pediatric patients, that Kt/V ≤ 1.3 was associated with low nPCR (1.01 ± 0.12 g/kg per day) and Kt/V > 1.3 with higher nPCR (1.27 to 1.33). Yet, increasing dialysis dose (Kt/V) beyond 1.6 did not have any further effect on nPCR. Tom and colleagues [34] employed an intensive program in which 12 children received an average of 155.9% of their recommended protein intake and aggressive urea clearance (Kt/V > 1.8) and showed increased height SDS in ten of their children. They concluded that aggressive nutritional support and enhanced dialysis delivery lead to improved growth. In two pediatric studies [20, 21], patients’ nutrition status as measured by nPCR increased (monthly mean 1.07 to 1.38) with IDPN, with no change in their dialysis dose (monthly mean of sp Kt/V 1.36 to 1.26). Studies of adults have shown that daily HD or nocturnal HD can improve appetite and food intake. This could contribute to increased sense of well being, fewer dietetic restrictions and decreased use of medications such as phosphate binders and sodium polystyrene sulfonate, drugs known to cause poor appetite. In a pilot pediatric study, Fischbach et al. [35] showed that hemodialysis five to six times a week led to increased growth velocity in five pediatric patients. The underlying mechanism for improved growth may not be attributed solely to increment of the dialysis dose, since the patients also received online hemodiafiltration with ultra-pure dialysate, which could limit inflammation. Moreover, dietary restrictions were removed for these children, allowing them to have a high protein intake of 2 g/kg to 2.5 g/kg. Thus, we suggest that increasing dialysis dose without addressing nutritional needs may not result in improved growth.

##### Nutrition provision

Recommended dietary allowance (RDA) is the daily dietary intake level of a nutrient considered sufficient to meet the requirements of nearly all (97–98%) healthy individuals in each life stage and gender group. The K-DOQI pediatric guidelines [22] recommend caloric requirement to meet the RDA for age, and initial dietary protein intake to meet RDA for chronological age and an additional increment of 0.4/g/kg per day to be provided to children receiving HD. This recommendation is based on studies of adults that documented protein losses in fasting patients, during high-flux hemodialysis, of approximately 8 g during a routine session [36] and for whom the ingestion of 1.1 g/kg per day of protein was not adequate to maintain nitrogen balance [37]. While it is clear that provision of nutritional supplement is the most important factor in treating malnutrition in infants and young children, this may not be true in older children. In fact, whether nutritional supplements benefit children over 2 years of age has been questioned [38]. Five studies addressed nutritional supplementation in older children with chronic kidney disease out of which two showed improvement in growth. However, none of these studies were primarily of children receiving HD [39–43]. Two pediatric studies suggested that increased caloric and protein intake in addition to optimal dialysis is needed to improve nutritional status (either growth or weight gain) in older children receiving HD [20, 34]. Meeting the special nutritional needs for these children requires nutritional supplement in the form of high calorie formulas by oral or tube feeding/G-tube feedings. Though the benefits of tube feeding for young children and infants are clear, there are no studies available that evaluate the success of this intervention in older children and adolescents receiving hemodialysis. In a systematic review of 28 studies of adults receiving hemodialysis, enteral nutritional supplements, either as oral or tube feeds, were shown to increase serum albumin by 2.3 g/l [44].

Intradialytic parenteral nutrition (IDPN) has the advantage of providing proteins and calories during HD without the need for a separate central venous catheter. The carbohydrate content prevents protein catabolism rather than meeting caloric needs. Numerous studies of adults have demonstrated the efficacy of this method [45, 46] in improving whole-body protein synthesis and a significant decrease in whole-body proteolysis whereby there is net change from an essentially catabolic state to positive protein balance. A few studies have looked at the effectiveness of IDPN in children. An early study showed no improvement in amino acid levels after supplementation with a low dose of amino acid (0.25 gm/kg) during dialysis [47]. Goldstein et al. [20] highlighted the effectiveness of IDPN by demonstrating reversal of weight loss and initiation of weight gain within 6 weeks of starting IDPN in three adolescent patients who had undergone ≥ 10% weight loss over a 3-month period. In that study 40% of weekly-prescribed protein intake was provided by IDPN (1.3 gm/kg per treatment). In a subsequent study by Orellana et al. [21], IDPN was instituted for all adolescent patients who had lost 10% body weight over a 3-month period and were at < 90% of their ideal body weight, irrespective of the presence or absence of gastrointestinal illness. The seven patients with organic illness gained weight or BMI during the first 5 months of IDPN therapy, whereas two patients with psychosocial-associated malnutrition did not [21], as shown in Table 2. The patients with psychosocial malnutrition might not have improved their enteral intake secondary to depression or might have had inadequate access to food after IDPN initiation, resulting in their lack of anthropometric improvement. We recommend IDPN be used for patients who have been losing weight of > 10% for 3 consecutive months and who are less than 90% of their ideal body weight and do not respond to enteral supplementation.

**Table 2:** IDPN and effect on BMI (patients 1 and 4 had psychosocial malnutrition, whereas, for the remaining patients, the causes were organic)

Patient	Duration (months)	BMI before IDPN	BMI after IDPN
1	14	17.3	15.1
2	8	21.7	23.4
3	16	20.3	22.4
4	12	19.2	17.3
5	22	20.1	22.7
6	5	17.2	20.4
7	11	19.2	20.8
8	3	17.5	17.7
9	5	15.3	16.2

In conclusion, optimizing maintenance hemodialysis in children not only rests ensuring adequate dialysis dose but also on the provision of adequate nutrition. Because of the multifactorial pathogenesis of malnutrition, a multidisciplinary effort that includes nutritional, pharmacological and metabolic interventions is required to prevent malnutrition. Recent studies in children have focused on such interventions and have added to our understanding to adequately evaluate, prevent and treat malnutrition in children with ESRD receiving maintenance hemodialysis.

**Questions**

(Answers appear following the reference list)

- The anthropometric risk factors associated with increased mortality in children with ESRD are all the following except
- Height SDS
- Extremes of BMI
- Growth velocity SDS
- Weight SDS
- Serum albumin as marker of nutritional status in children has the following limitations except
- Long half-life
- Decrease in albumin level correlates with mortality
- Decreases with inflammation
- Decreases with fluid overload
- All the following statements are true about nPCR except
- Is a mathematical formula obtained from formal urea kinetic modeling
- Is estimated from urea generation rate
- Has recently emerged in children with ESRD receiving chronic hemodialysis as a marker of protein intake
- Was shown to be inferior to serum albumin as a marker of subsequent weight loss in recent studies
- Intradialytic parenteral nutrition (IDPN) has all the following advantages except
- Serves primarily as a protein source to patients receiving chronic hemodialysis
- Provides nutrition during hemodialysis without the need for a separate central venous catheter
- Reverses weight loss in all causes of protein energy malnutrition (PEM)
- Carbohydrate content prevents catabolism rather than meeting caloric needs
